# Endovascular treatment of a Superior Mesenteric Artery Syndrome variant secondary to traumatic pseudoaneurysm

**DOI:** 10.1186/1749-7922-5-7

**Published:** 2010-03-08

**Authors:** Iain Au-Yong, Nicholas FS Watson, Catherine L Boereboom, Timothy E Bowling, John F Abercrombie, Simon C Whitaker

**Affiliations:** 1 Department of Radiology, Nottingham University Hospitals NHS Trust, Derby Road, Nottingham, NG7 2UH, UK; 2 Department of Gastrointestinal Surgery, Nottingham University Hospitals NHS Trust, Derby Road, Nottingham, NG7 2UH, UK; 3 Clinical Nutrition Unit, Nottingham University Hospitals NHS Trust, Derby Road, Nottingham, NG7 2UH, UK; 4 Department of Surgery, Royal Derby Hospital, Uttoxeter Road, Derby, DN22 3NE, UK

## Abstract

Pseudoaneurysms related to the superior mesenteric artery (SMA) are a recognised complication of trauma to the vessel, and successful treatment with stenting has been previously described. We report the case of a patient who presented with obstruction of the fourth part of the duodenum secondary to a traumatic pseudoaneurysm, a hitherto unreported variant of superior mesenteric artery syndrome. Exclusion of the pseudoaneurysm and relief of the duodenal obstruction were simultaneously achieved by placement of a covered stent.

## Background

Superior mesenteric artery pseudoaneurysm is a rare but recognised complication of traumatic injury to the artery [1-8]. It is caused by a full thickness breach of the artery wall. Other recognised causes include pancreatitis and iatrogenic events. It may also occur spontaneously. The condition is important as the risk of rupture is high and carries a significant mortality rate [1].

Superior mesenteric artery syndrome is more widely recognised, and results from obstruction of the duodenum where it passes between the superior mesenteric artery and aorta, by any process which narrows the angle between these two structures [9]. In its commonest form it is not associated with an acquired structural abnormality: the angle between the SMA and aorta is constitutionally narrowed. In its best-known acquired variant, the aortoduodenal syndrome, the duodenum is compressed between the SMA and an abdominal aortic aneurysm [10]. This case is unique, comprising both the first description of a variant of SMA syndrome caused by a traumatic SMA pseudoaneurysm and the first account of successful treatment of both the aneurysm and duodenal obstruction by endovascular stent placement.

## Case Report

Our 40 year-old male patient was the driver of a vehicle that collided at high speed with a fence post. He was transferred via air ambulance to hospital and on arrival was conscious and alert. Marked anterior abdominal wall bruising was evident consistent with injury relating to use of a lap belt, and he complained of diffuse abdominal pain. Abdominal computerised tomography (CT) demonstrated free intraperitoneal fluid. At laparotomy, approximately 3000 mls of haemoperitoneum was evacuated and devascularising mesenteric injuries were noted affecting segments of jejunum, terminal ileum, caecum and sigmoid colon (American Association for the Surgery of Trauma Grade 4 injuries). A subtotal colectomy with ileo-sigmoid anastamosis and resection of 10 cm of mid-jejunum was performed.

**Figure 1:**
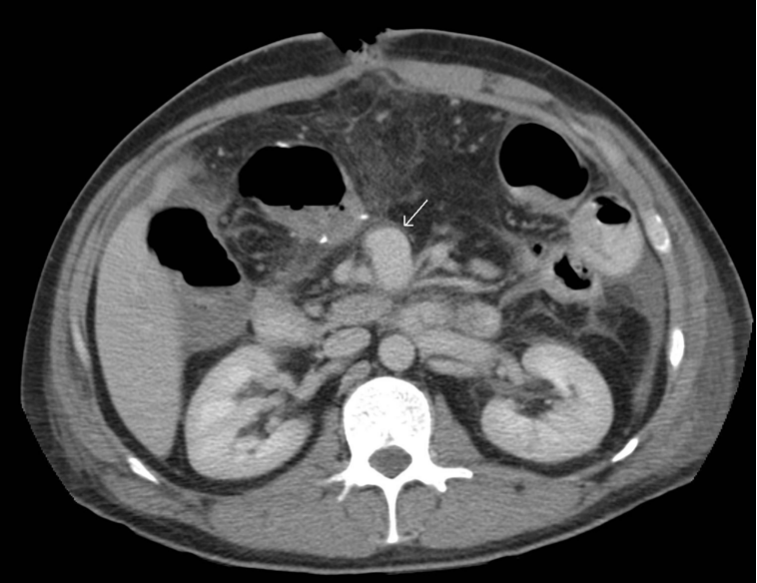
**Axial CT image with oral contrast demonstrating a small pseudoaneurysm (arrow) to the right of the SMA**.

**Figure 2:**
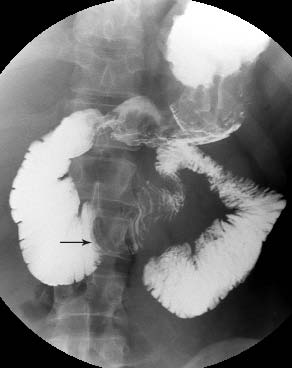
**Barium small bowel meal demonstrates dilatation of the first to third parts of the duodenum and a rounded filling defect at the level of the fourth part (see arrow)**.

**Figure 3:**
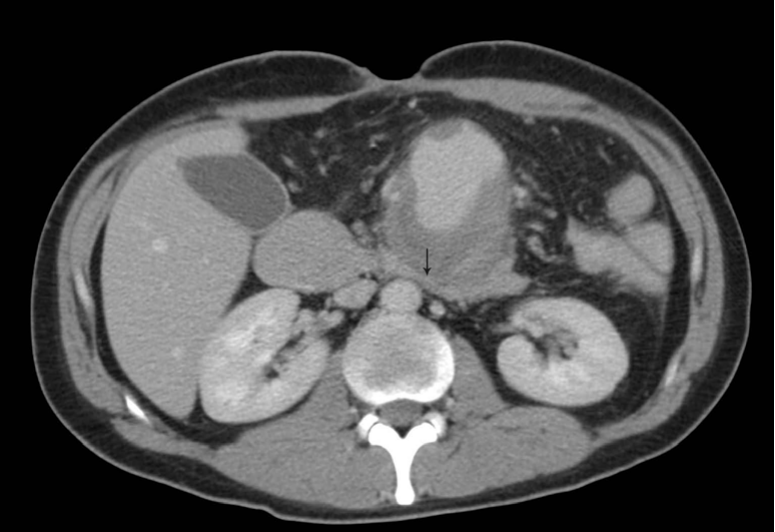
**Axial CT images demonstrating the SMA pseudoaneurysm compressing the fourth part of the duodenum (arrow)**.

**Figure 4:**
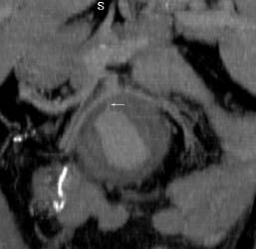
**3-dimensional reconstructions of the CT better demonstrating the anatomical relationships and demonstrating communication between the connection between the SMA and the aneurysm sac (arrow)**.

Postoperative recovery was prolonged due to persistent vomiting, initially thought to be secondary to ileus. CT performed on postoperative Day 12 showed small bowel dilatation consistent with ileus and the small bowel anastomosis appeared unremarkable. This also demonstrated a small aneurysm at the SMA origin, which was only appreciated in retrospect (Figure 1). The presence of oral contrast opacifying most of the small bowel made interpretation more difficult. Two weeks later a barium small bowel meal was performed due to persistent nausea and vomiting. This examination demonstrated dilatation of the proximal duodenum, with hold up of barium to the level of the fourth part, where a rounded filling defect causing extrinsic compression was noted (Figure 2). The patient subsequently became acutely unwell with a fever of 39.3°C, leucocytosis and tachycardia. A differential diagnosis of central venous catheter-related sepsis or intra-abdominal collection was considered and another abdominal CT was performed (two days after the small bowel meal). This demonstrated a 6.3 cm pseudoaneurysm in the central abdomen intimately related to the superior mesenteric artery (Figures 3 and 4). In addition, the stomach and duodenum were dilated, with narrowing of the fourth part of the duodenum caused by extrinsic compression by the aneurysm sac. Oral contrast in this case was held up at the level of the obstruction. Blood cultures taken from the patients indwelling central venous catheter grew a sensitive staphylococcus aureus, and the sepsis resolved with removal of the infected catheter.

**Figure 5:**
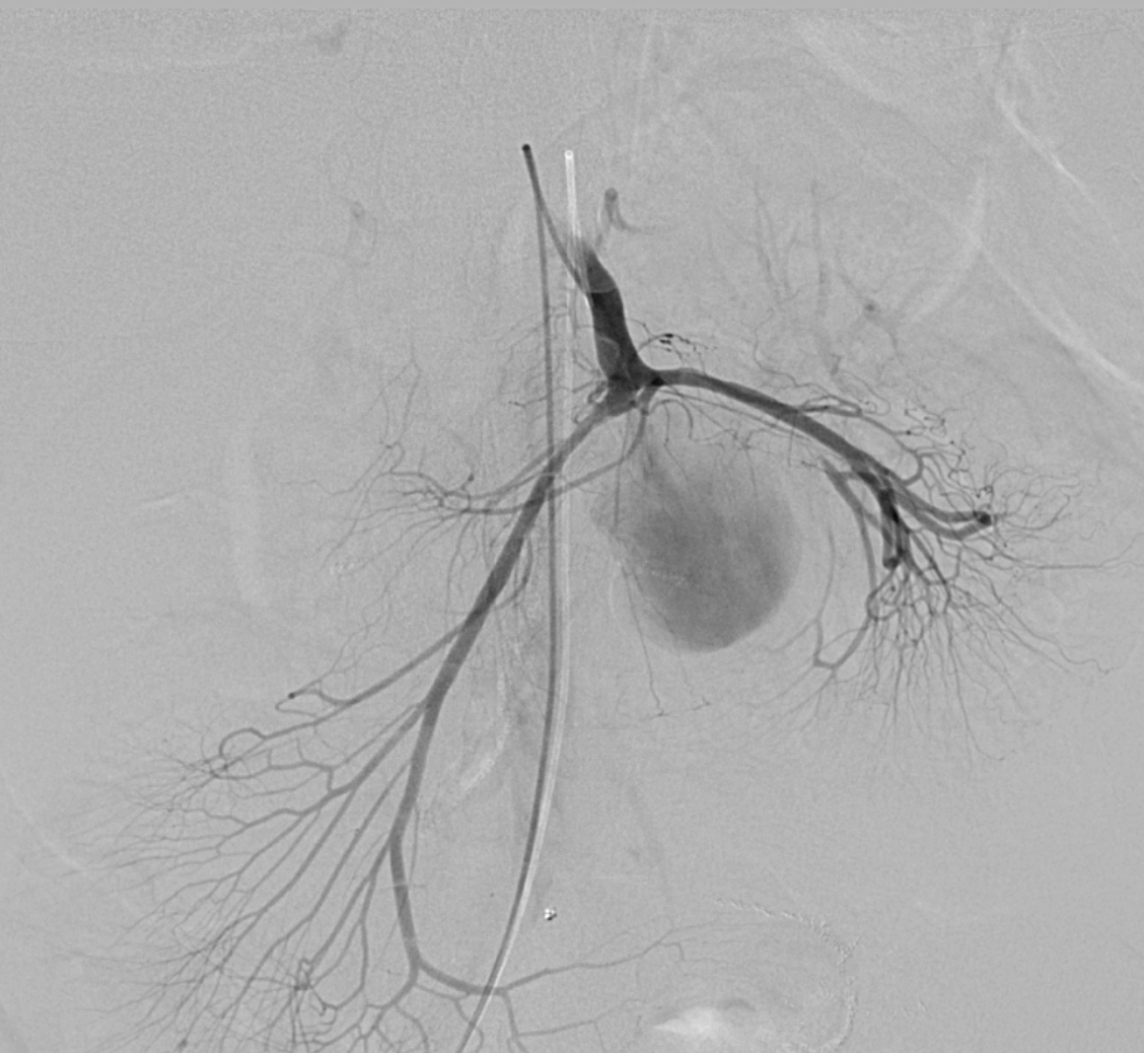
**Angiographic images from which the size of the defect into the pseudoaneurysm can be appreciated**.

The potential risks of surgical repair of the pseudoaneurysm were considered to be very high for this patient, therefore mesenteric angiography was undertaken with a view to endovascular management. Selective angiography confirmed a large pseudoaneurysm arising from the main stem of the SMA, just beyond its first major jejunal branch (Figure 5). The aneurysm had no distinct neck and the vessel wall defect appeared to be substantial. Splayed vessels were noted draped around the pseudoaneurysm. Of the potential endovascular therapeutic options, embolisation and thrombin injection both risked occlusion of all or part of the SMA territory and were considered unsuitable whereas placement of a covered stent provided an opportunity to exclude the aneurysm without loss of the main vessel lumen.

**Figure 6:**
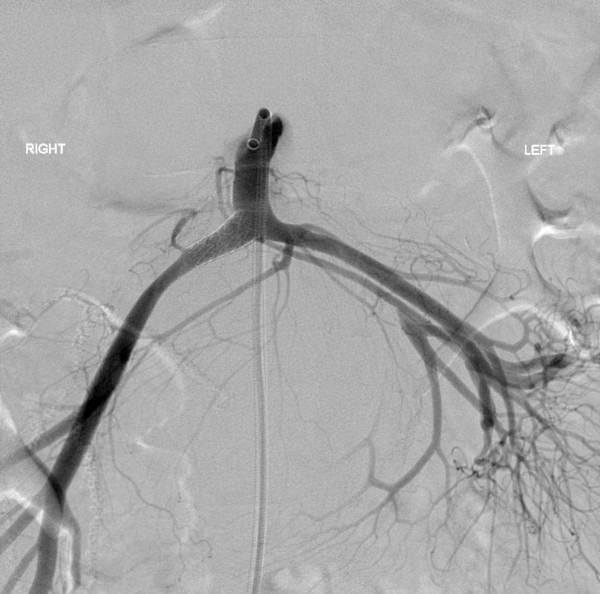
**Angiographic image demonstrating appearances post-stent placement**.

**Figure 7:**
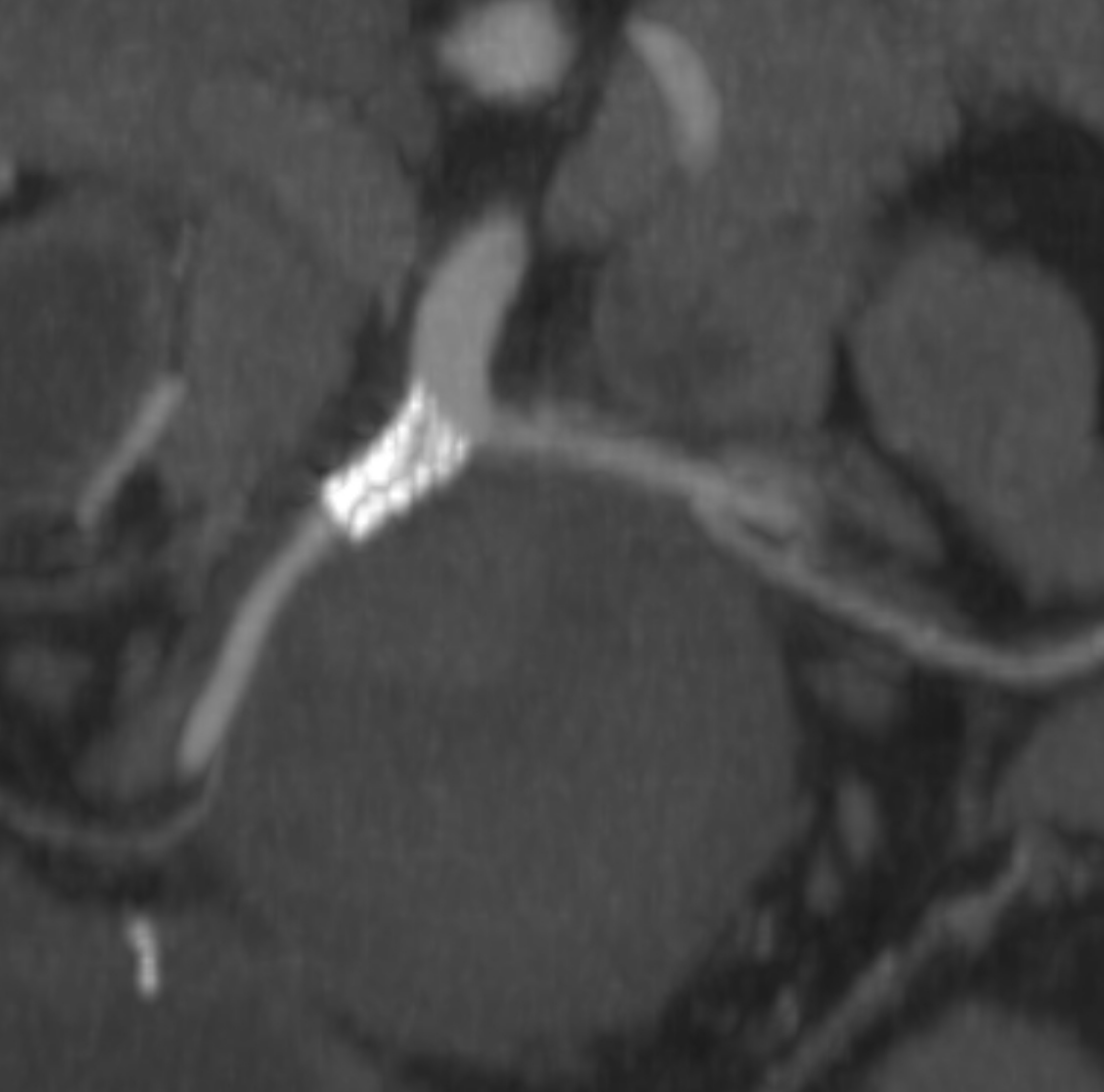
**3-dimensional reconstruction demonstrating exclusion of the aneurysm following placement of the stent within the SMA**.

A 6F guiding sheath (Destination, Terumo Corporation) was advanced into the SMA and past the aneurysm, over a stiff hydrophilic wire (Terumo, Terumo corporation). A 5 mm diameter × 16 mm length covered Palmaz stent (Atrium V12) was then deployed across the mouth of the aneurysm. Because of the difference in diameter of the SMA proximal and distal to the aneurysm origin, the proximal half of the stent was flared by dilatation with a 7 mm angioplasty balloon (Cordis). Although angiography at this stage showed no leak (Figure 6), a subsequent CT angiogram demonstrated persistent perfusion of the sac. The proximal half of the stent was therefore dilated further, using an 8 mm angioplasty balloon (Cordis) at a second procedure. Follow-up CT angiography confirmed successful exclusion of the aneurysm (Figure 7).

The patient reported an almost immediate improvement in nausea and vomiting on clinical review on the ward following the first procedure and was discharged home. He did not attend hospital for subsequent follow-up imaging, but on telephone review remains well one year post-procedure with no recurrence of any of his symptoms.

In this case, follow up imaging would have been useful to examine for involution of the pseudoaneurysm and continued exclusion, as well as resolution of splaying of the vessels.

## Discussion

This unique case comprises both the first description of a variant of SMA syndrome caused by a traumatic SMA pseudoaneurysm, and the first account of successful treatment of both the aneurysm and duodenal obstruction by endovascular stent placement. Two similar cases were described in 1990 [11], however, in these cases, obstruction was caused by rupture of an SMA pseudoaneurysm, treated with open surgery.

Barium meal examination is useful for the diagnosis of SMA syndrome [9]. It demonstrates both narrowing of the fourth part of the duodenum with increased transit time, proximal dilatation and uncoordinated peristaltic activity. Such functional information is not readily obtainable from CT.

CT proved to be the key modality for diagnosis in this patient. It enabled detection of the pseudoaneurysm and its relationship to the SMA. CT with 3D reconstruction has been used in SMA syndrome to demonstrate reduction of the angle between the SMA and the aorta [12].

Despite the paucity of cases of SMA pseudoaneurysm, several reports describe successful endovascular treatment of this condition. Open surgery is often rendered difficult by the underlying cause of the psuedoaneurysm (such as pancreatitis) or by adhesions, which increase the risk of failure of open vascular reconstruction and of anaesthesia in the unstable patient [1]. Other options for treatment of this condition include placement of coils, injection of thrombin or N-butyl-2-cyanoacrylate (glue) [1].

This case presented an unusual challenge, as two problems needed addressing; stenting of the aneurysm to prevent subsequent rupture, and exclusion of the aneurysm sac to encourage involution and thus relieve the SMA syndrome. The immediate resolution of this patient's symptoms was most likely due to loss of pressure within the aneurysm sac by exclusion of arterial inflow. Data on possible shrinkage of aneurysm sacs post-stenting are conflicting, with one large series of 90 endovascular repairs of a range of visceral artery aneurysms demonstrating no shrinkage at follow-up imaging [1]. However, one study reported shrinkage of abdominal aortic aneurysms post-stent placement [13]. This phenomenon, in addition to decreased pressure within the sac, may be helpful in the treatment of aortoduodenal syndrome, which has hitherto only been treated by open repair.

## Conclusions

A unique case of a variant of SMA syndrome secondary to a pseudoaneurysm is presented. Exclusion of the aneurysm and relief of the obstruction were simultaneously achieved by placement of a stent.

## Consent

Written informed consent was obtained from the patient for publication of this case report and any accompanying images. A copy of the written consent is available for review by the Editor-in-Chief of this journal.

## Competing interests

The authors declare that they have no competing interests.

## Authors' contributions

All authors participated in the conception, design, data collection and interpretation, manuscript preparation and literature search.
